# Gyroscopic Radiosurgery for Trigeminal Neuralgia: Results of a Retrospective Series of >50 Patients

**DOI:** 10.3390/brainsci16101061

**Published:** 2026-09-30

**Authors:** Luis Real Peña, Morena Sallabanda, Marta Quirós Martí, Kita Sallabanda

**Affiliations:** 1 Hospital Clínico Universitario de Valencia, 46010 València, Spain; mquirosmarti@gmail.com; 2 Instituto de Radiocirugía Avanzada (IRCA), 28003 Madrid, Spain; ksallabanda@irca-radiocirugia.com

**Keywords:** trigeminal neuralgia, stereotactic radiosurgery, gyroscopic radiosurgery, ZAP-X, facial pain, Barrow Neurological Institute, radiosurgery, treatment outcome

## Abstract

**Highlights:**

**What are the main findings?**

- Gyroscopic radiosurgery provides high rates of pain control in refractory Trigeminal Neuralgia (87.5%).
- Treatment-related toxicity occurred in 8.9%.

**What are the implications of the main findings?**

- Gyroscopic radiosurgery is a non-invasive, safe and effective radiosurgical alternative for the treatment of Trigeminal Neuralgia.
- Half of the patients who experienced clinical improvement achieved it within the first 15 days.

**Abstract:**

Background: Stereotactic radiosurgery (SRS) is an established treatment option for medically refractory trigeminal neuralgia (TN). However, clinical evidence regarding dedicated gyroscopic radiosurgery systems remains limited. This study aimed to evaluate the efficacy, latency of response, safety, retreatment rate, and potential clinical and dosimetric predictors of outcome following ZAP-X gyroscopic radiosurgery for TN. Methods: A consecutive series of patients with pharmacologically refractory TN treated with ZAP-X gyroscopic radiosurgery between February 2023 and September 2025 was analyzed. Treatment consisted of a single isocenter targeting approximately 5 mm of the cisternal-retrogasserian trigeminal nerve, with a maximum dose of 90 Gy and a V70 Gy coverage objective ≥ 50%, using 4 or 5 mm collimators. Pain outcome was assessed using the Barrow Neurological Institute Pain Intensity Scale (BNI), and sensory toxicity was evaluated using the BNI Facial Numbness Scale. Clinical and treatment-related predictors of failure and retreatment were explored using binary logistic regression. Results: Following radiosurgery, 87.5% of patients achieved satisfactory pain control (BNI I–IIIb; 95% CI, 78.8–96.2%), with a significant overall improvement in BNI score compared with baseline (*p* < 0.001) at the 12 months of follow-up. Half of the patients experienced clinical improvement within the first 15 days. Treatment-related toxicity occurred in 8.9% of patients, while 26.8% required repeat radiosurgery during follow-up. The decision to perform retreatment is made when the patient has shown no improvement in the BNI score at 6 months after the initial treatment. Baseline BNI grade V was the only statistically significant predictor of treatment failure, conferring a more than ninefold higher odds of non-response compared with BNI grade IV (OR 9.33; 95% CI, 1.66–52.3; *p* = 0.011). Female sex showed a nonsignificant trend toward a lower risk of failure (OR 0.65; 95% CI, 0.13–3.24; *p* = 0.596). Neither multiple sclerosis-associated TN nor other etiological or dosimetric variables significantly predicted treatment failure. A greater number of beams showed a trend toward an increased probability of retreatment (OR 1.01; *p* = 0.091), although statistical significance was not reached. Conclusions: Gyroscopic radiosurgery with ZAP-X provides high rates of pain control, rapid clinical response, and a favorable safety profile in patients with refractory TN. Greater baseline pain severity appears to be associated with poorer treatment response. These findings support ZAP-X as an effective frameless radiosurgical alternative for TN, although larger prospective studies are required to identify robust clinical, dosimetric, and radiobiological predictors of long-term outcome.

## 1. Introduction

Trigeminal neuralgia (TN), historically described as tic douloureux, is a paroxysmal facial pain syndrome whose diagnosis remains fundamentally clinical [1]. According to the International Classification of Headache Disorders, 3rd edition (ICHD-3), it is characterized by recurrent episodes of unilateral, brief, electric shock-like pain, with abrupt onset and termination, limited to one or more divisions of the trigeminal nerve and usually triggered by innocuous stimuli [2]. The current classification distinguishes classical, idiopathic, and secondary forms, a distinction that is relevant from both pathophysiological and therapeutic perspectives [2,3]. Its global incidence is 4 to 5 cases per 100,000 population [2].

Pharmacological treatment constitutes first-line therapy. In patients with refractory pain or intolerance to medical treatment, stereotactic radiosurgery (SRS) represents an established alternative, with extensive clinical experience, particularly with Gamma Knife radiosurgery (GKRS) [3,4]. Most of the historical evidence comes from Co-60-based systems and procedures performed with stereotactic frames; subsequently, the incorporation of image-guidance systems enabled frameless strategies using LINAC and robotic platforms while maintaining the precision required for treatment of the trigeminal nerve [5,6].

ZAP-X (ZAP Surgical Systems, Inc., San Carlos, CA, USA) is a dedicated gyroscopic radiosurgery system that integrates a 3-MV linear accelerator mounted on two rotational axes, with a source-to-isocenter distance of 45 cm and angular coverage exceeding 2π steradians. The beam is collimated using circular apertures ranging from 4 to 25 mm and can be delivered at a nominal rate of up to 1500 MU/min [7]. These characteristics allow the generation of non-coplanar beams with steep dose gradients, of particular interest for irradiation of small-volume trigeminal segments [7,8]. Romanelli et al., in studies published in 2003 and 2018, demonstrated that the accuracy required for this procedure could be achieved using thermoplastic masks and intraprocedural image-guidance systems. This development ultimately made it possible to treat trigeminal neuralgia using gyroscopic and non-gyroscopic LINAC platforms [5,6].

Several clinical variables related to the patient’s treatment history and radiosurgical planning have been associated with the likelihood of achieving adequate initial pain control after radiosurgery. Advanced age is among the factors associated with a more favorable response, with better outcomes reported particularly in patients aged 70 years or older [9]. Furthermore, a typically paroxysmal pain pattern, characteristic of classical trigeminal neuralgia, appears to be associated with a higher probability of response than forms with a continuous or atypical pain component [9].

Conversely, certain aspects of the patient’s history may predict a less favorable response. In some series, patients who had previously undergone trigeminal surgery were less likely to maintain satisfactory pain control [10]. Among these procedures, previous ablative treatments, particularly radiofrequency thermocoagulation, have been associated with a significant reduction in radiosurgical efficacy [11]. More specifically, a history of microvascular decompression (MVD) has also been associated with a lower probability of achieving initial pain cessation after Gamma Knife treatment [12].

The clinical characteristics of pain also appear to have prognostic value. The presence of an atypical or continuous pain component has been associated with poorer analgesic outcomes [9,10], as has a pre-existing trigeminal sensory deficit [10]. In the latter setting, the absence of preoperative sensory abnormalities was significantly associated with a higher probability of sustained pain relief (*p* = 0.02) [10].

Age may also act as a prognostic variable: whereas age ≥ 70 years has been associated with a higher probability of pain control, younger patients, with thresholds ranging from 60 to 70 years across studies, appear to have slightly less favorable outcomes [9]. However, this association has not been consistently reproduced in all studies.

Among technical planning variables, the position of the isocenter relative to the point at which the trigeminal nerve emerges from the brainstem appears to be particularly relevant. A distance greater than 8 mm has been associated with poorer pain control. In a series of 112 patients, the cumulative rate of pain control without medication at 6 months was 52.9% when the distance was ≤8 mm, compared with 25% when it exceeded 8 mm (*p* = 0.017). This difference persisted over the long term, with 5-year pain-control rates of 70% and 26%, respectively (*p* < 0.001) [13].

Finally, certain comorbidities may modify the response. Diabetes mellitus has been associated with lower Gamma Knife efficacy, with an odds ratio for a favorable response of 0.38 (*p* = 0.013) [11]. Although multiple sclerosis has traditionally been considered an unfavorable prognostic factor, more recent evidence qualifies this association. A comparative study found no significant difference in the initial response between patients with and without multiple sclerosis (77% vs. 69%), but reported more frequent recurrence and a significantly shorter duration of analgesic effect among patients with multiple sclerosis [14]. It therefore appears more appropriate to regard multiple sclerosis as a factor associated with less durable pain control rather than as an unequivocal predictor of initial treatment failure.

The specifically published experience with ZAP-X in TN remains limited, although the initial series have confirmed the technical feasibility of the procedure and reported favorable early clinical outcomes [8,15]. In this context, the present study analyzes a consecutive cohort of patients with TN treated with gyroscopic radiosurgery, with the aim of evaluating analgesic response, its latency and durability, recurrence, sensory toxicity, and the potential influence of clinical and dosimetric variables on outcomes.

The primary endpoint was pain response at 6 months after gyroscopic radiosurgery, assessed using the Barrow Neurological Institute Pain Intensity (BNI-PI) scale. A favorable clinical response was defined as BNI grades I–IIIa, whereas BNI grades IIIb–V were considered treatment failure. Patients showing no improvement in BNI grade at 6 months were considered eligible for retreatment according to the institutional treatment protocol.

Secondary endpoints included latency to pain relief, defined as the interval between radiosurgery and the first documented improvement in BNI grade; magnitude of pain response; durability of pain control; recurrence after an initial favorable response; retreatment rate; and treatment-related sensory toxicity. Recurrence was defined as deterioration to BNI IIIb–V after a previous favorable response (BNI I–IIIa). Sensory toxicity was evaluated according to the occurrence and severity of new facial numbness, paresthesia, dysesthesia, or bothersome hypoesthesia following treatment.

Exploratory endpoints included the association between clinical and treatment-related variables and pain response, recurrence, retreatment, and sensory toxicity. Clinical variables included patient demographics, trigeminal neuralgia etiology and phenotype, disease duration, previous surgical or ablative procedures, and baseline neurological status. Treatment-related variables included dosimetric and technical parameters such as prescription dose, nerve coverage, V70, number of arcs, treatment duration, and radiation exposure to adjacent organs at risk.

## 2. Materials and Methods

### 2.1. Study Population, Clinical Variables, and Follow-Up

Between 1 February 2023 and 1 September 2025, 56 patients with TN were retrospectively treated with ZAP-X gyroscopic radiosurgery; the patients were treated before 1 September 2025, not adding patients with posterior treatment. Patients with pharmacologically refractory TN were included irrespective of etiology, classified according to ICHD-3 [2]. For the purposes of this retrospective study, pharmacologically refractory TN was defined as trigeminal neuralgia fulfilling ICHD-3 diagnostic criteria in which adequate pain control could not be achieved with first-line pharmacological treatment, or in which such treatment could not be continued because of clinically relevant adverse effects. First-line therapy consisted primarily of sodium-channel-blocking agents, namely carbamazepine and/or oxcarbazepine, in accordance with standard clinical practice and current guidelines. Age, sex, date of diagnosis, etiology, previous treatments, and clinical course were recorded. Pain was assessed using the Barrow Neurological Institute Pain Intensity Scale (BNI), and sensory toxicity using the BNI Facial Numbness Scale. Follow-up was scheduled at approximately 15, 30, 90, and 180 days and was subsequently individualized according to clinical evolution. Response was defined as BNI I–IIIb and recurrence as reappearance of BNI IV–V pain after an initial response. Medication was progressively reduced according to clinical control. Hypoesthesia, bothersome hypoesthesia, dysesthesia, and corneal abnormalities were specifically recorded. In the absence of response or in the event of recurrence from 6 months onward, a second irradiation to an adjacent trigeminal segment was considered, while maintaining the prescription and organ-at-risk protection criteria.

### 2.2. Image Acquisition, Registration, and Target Definition

Treatment planning was based on cranio-cervical CT and 1.5-T MRI acquired in the supine position with thermoplastic mask immobilization. Planning CT (Philips Ingenuity, Philips, Singapore) was acquired with a slice thickness of ≤1 mm and was used as the primary dataset for dose calculation. MRI (Philips Ingenia, 1.5 T, Philips, Singapore) included 3D T1 and T2 sequences, contrast-enhanced T1, and a high-resolution 3D T2 sequence for visualization of the cisternal-retrogasserian course. Images were registered in Brainlab Elements using rigid and, when necessary, deformable fusion, with visual anatomical verification. The target encompassed approximately 5 mm of the cisternal-retrogasserian segment of the trigeminal nerve. The exact location of the radiosurgical target within the cisternal-retrogasserian segment of the trigeminal nerve was individualized for each patient. Target selection was based primarily on the anatomical relationship between the nerve and the brainstem and on the available length of the cisternal trigeminal segment. When sufficient nerve length was available, a more distal retrogasserian target was generally preferred to increase the distance from the brainstem and limit exposure of the pontine surface to intermediate and high isodoses. Conversely, in patients with a shorter cisternal segment or anatomical constraints that limited distal targeting, a more proximal cisternal location was selected while maintaining the predefined brainstem dose constraints. Therefore, target location was not assigned according to a fixed anatomical distance, but rather individualized according to each patient’s trigeminal anatomy and the dosimetric feasibility of achieving adequate nerve coverage while minimizing brainstem irradiation. The brainstem was contoured as an organ at risk, together, depending on proximity, with brain parenchyma, temporal lobe, optic pathway, globes, and cochleae.

### 2.3. Treatment Planning and Dosimetric Prescription

Volumes were exported to the ZAP-X Treatment Planning System (version 1.10.1.16236). Dose calculation was performed using the ray-tracing algorithm, and planning combined direct isocenter positioning with inverse beam optimization. A single isocenter was used over the selected segment, with a maximum dose of 90 Gy prescribed to 100% and a coverage objective of V70 Gy ≥ 50%; 4 or 5 mm collimators were used. A 4 mm collimator was used in the majority of patients, consistent with the most commonly employed aperture in the radiosurgical treatment of trigeminal neuralgia and with the small caliber of the cisternal trigeminal nerve. In the most recently treated patients, however, a 5 mm collimator was preferentially selected. This modification reflected an evolution in our institutional planning strategy prompted by cine-MRI studies suggesting that the trigeminal nerve may exhibit measurable physiological motion. Although the clinical relevance and magnitude of this motion remain incompletely characterized, these observations raised the possibility that very narrow irradiation fields could be more sensitive to geometric uncertainties. The 5 mm collimator was therefore introduced as a pragmatic strategy to provide a modestly wider high-dose envelope around the selected nerve segment and potentially reduce the impact of motion-related targeting uncertainty, while maintaining the steep dose gradients required to limit irradiation of adjacent structures. Because collimator selection was not randomized and changed over time, no causal inference regarding superiority of either collimator size can be made from the present cohort. For the brainstem, priority was given to V12 Gy < 0.2 cm^3^ and V10 Gy < 0.5 cm^3^. All plans were jointly reviewed by neurosurgery, radiation oncology, and medical physics, assessing the three-dimensional isodose distribution, coverage, and dose-volume histograms. The reported irradiated trigeminal nerve volume was defined as the volume of the contoured trigeminal nerve encompassed by the 90-Gy prescription isodose. Additional target and organ-at-risk dose-volume parameters were retained within the individual ZAP-X treatment plans but were not incorporated into the anonymized research database.

Detailed treatment-planning images, dose-volume histograms, and organ-at-risk dosimetric parameters remained linked to identifiable clinical records and were not included in the anonymized research database. Their retrospective extraction for research purposes would have required additional ethical and data-protection review under Spanish regulations; consequently, these data were not incorporated into the present analysis.

### 2.4. Verification and Delivery

Correct alignment was verified before irradiation and intrafractionally at each gantry position by comparing the acquired images with digitally reconstructed radiographs generated from the planning CT. Automatic realignment was performed if deviations were detected. The nominal delivery rate was 1500 MU/min, and an ionization chamber monitored the emitted radiation in real time.

### 2.5. Statistical Analysis

The study collected data on disease diagnosis, technical characteristics of the irradiation process, pre- and post-treatment pain assessment, time to response, toxicity, and the need for retreatment.

Pain assessment was formalized using the BNI scale and constituted the primary study endpoint. It was measured before (T0) and after radiation and follow up of 12 months of the patients (T1). For statistical analysis, it was also converted into a binary variable:

- Success (responders): grades I to IIIb;
- Failure (non-responders): grades IV and V.

Descriptive analysis provided the most relevant statistics for all variables collected in the study: absolute and relative frequencies (for categorical variables), and mean, standard deviation, range, median, and quartiles (for continuous variables).

Regarding inferential analysis:

Simple binary logistic regression models were estimated to explain the probability of failure (or success) as a function of each patient- and intervention-profile variable. Unadjusted ORs and 95% confidence intervals were obtained from the Wald Chi-square statistic. Variables that were significant (*p* < 0.05) or relevant (*p* < 0.1) were to be incorporated into a final multivariable model (adjusted ORs). No final multivariable logistic regression model was ultimately performed. The statement indicating that variables meeting predefined univariable significance thresholds “were to be incorporated” into a multivariable model referred to the initially planned analytical strategy. However, only one variable reached statistical significance in the univariable analyses. Consequently, there was no meaningful set of candidate variables to enter into a multivariable model. In addition, once the final number of treatment-failure events was established (n = 7), construction of a conventional adjusted multivariable model was considered statistically inappropriate because of the high risk of overfitting and unstable coefficient estimates.

Accordingly, the analyses actually reported in the manuscript are univariable logistic regression models only, providing unadjusted odds ratios and 95% confidence intervals. The predefined *p*-value thresholds mentioned in the Methods were therefore not ultimately used for data-driven variable selection into a final adjusted model, as only a single variable fulfilled the significance criterion and the limited number of events precluded reliable multivariable modelling.

The same model was used to study the outcome ‘need for retreatment’.

The significance level used in the analyses was 5% (α = 0.05).

A post hoc calculation concluded that, with the current sample, statistical power would be 50% to detect failure rates of 5% and 25% as significantly different between two groups, at a 95% confidence level.

Only very large differences in failure rates (5% vs. 35%) would yield acceptable statistical power (=73.8%) with the current sample.

## 3. Results

**Figure 1:**
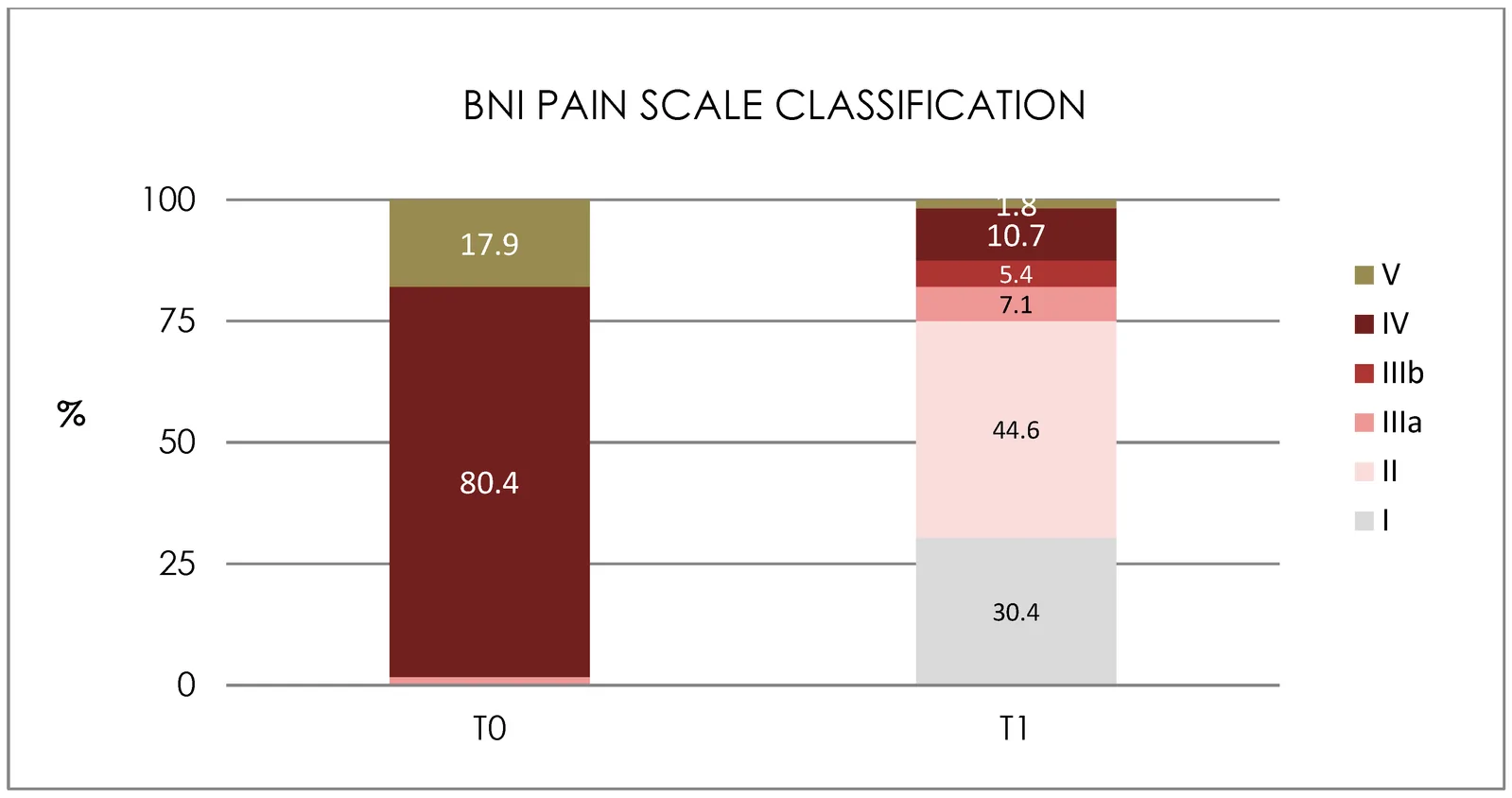
Distribution of pain according to the BNI scale before treatment (T0) and after treatment (T1). As can be observed, only 17.9% of patients experienced treatment failure following the procedure, defined as a BNI score of IV, or V, whereas the remaining 82.1% achieved a favorable therapeutic response.

Figure 1 describes the patients’ pain classification before and after treatment.

Therefore, at baseline, all patients except one had grade IV or V pain. After treatment, only 12.5% did. The final success rate (BNI grade < IV) was 87.5%, with a 95% confidence interval of 78.8–96.2%.

No patient worsened. At most, patients remained at the same grade (4 cases):

▪ 3 patients initially with grade IV remained at grade IV after the procedure.
▪ 1 patient initially with grade V remained at grade V after the procedure.

The remaining 52 patients improved by at least one grade on the scale.

The Stuart–Maxwell test confirmed a statistically significant overall improvement (*p* < 0.001).

**Figure 2:**
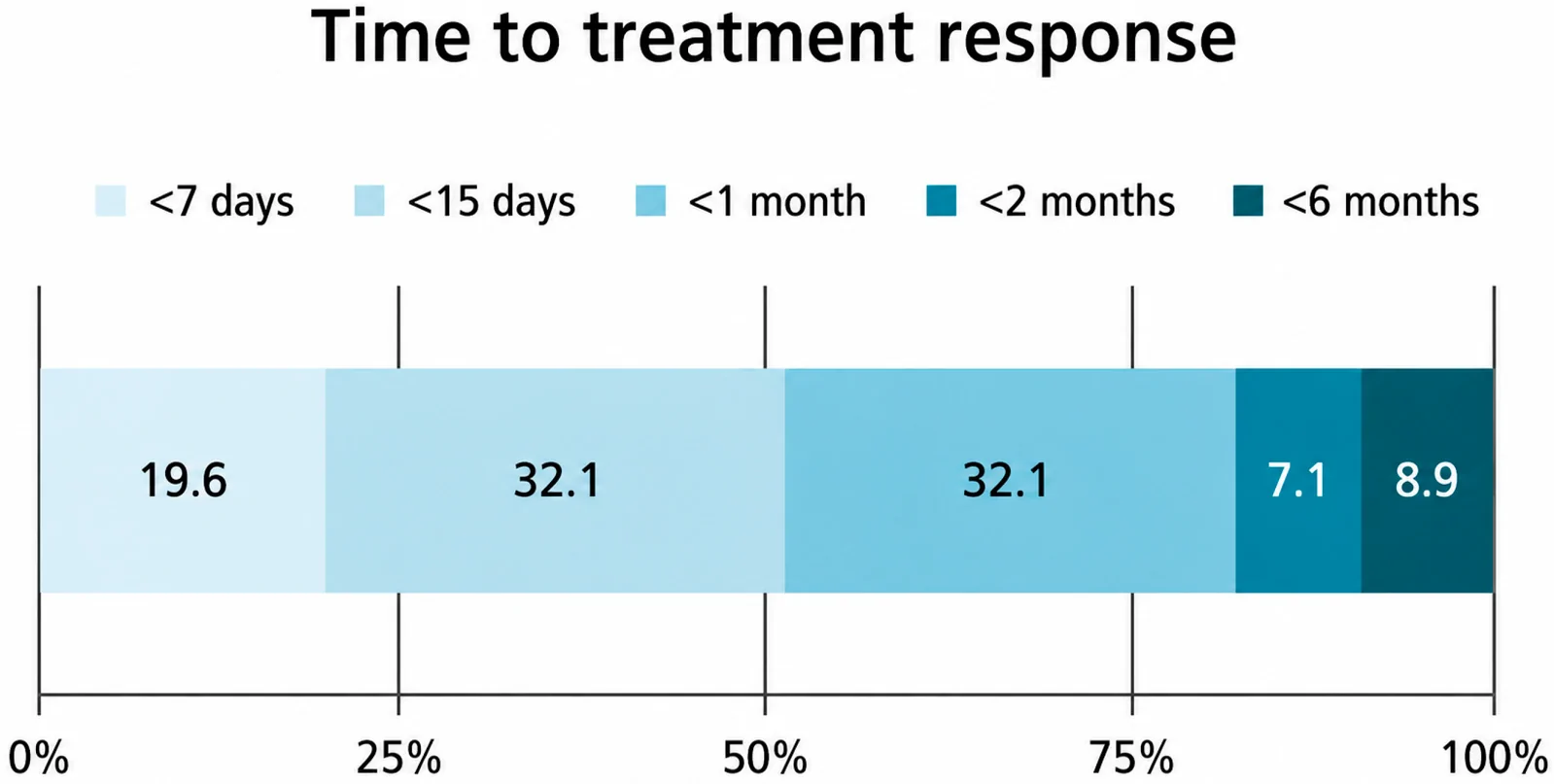
Analysis of time to treatment response.

Regarding secondary outcomes, half of the patients experienced improvement during the first 1 month after radiation, as reflected in Figure 2.

The toxicity rate, i.e., the proportion of patients with adverse effects, was 8.9% (5 cases). Treatment-related sensory toxicity was observed in five patients (8.9%). Four patients developed BNI Facial Numbness Scale grade II toxicity, corresponding to mild facial numbness that was not bothersome, while one patient developed grade III toxicity, corresponding to somewhat bothersome facial numbness. All sensory disturbances were transient and had completely resolved by the 6-month follow-up visit. No corneal complications were observed in any patient.

Up to 26.8% of patients required retreatment (15 cases).

As explained above, the failure rate was 12.5%, i.e., the proportion of patients with uncontrolled pain after the intervention.

The purpose of this section is to investigate which demographic and clinical patient factors and/or intervention-related factors predispose to failure (Table 1).

Among men, the failure rate was 15.8%, compared with 10.8% among women. In other words, female sex descriptively reduced the risk of failure (Table 2). Female sex was associated with an unadjusted OR for treatment failure of 0.65 (95% CI 0.13–3.24; *p* = 0.596); however, no statistically significant association between sex and treatment outcome was demonstrated. To assess the effect of sex and the other factors, binary logistic regression models were estimated. Failure (non-response to treatment) according to patient profile and intervention characteristics is reflected in Table 3.

Only baseline pain significantly influenced the final grade (OR = 9.33; *p* = 0.011): a patient with baseline grade V had more than 9 times the odds of failure compared with a patient with grade IV (Table 4).

Forty percent of patients who started with grade V ultimately failed to achieve pain control, compared with only 6.7% of those who started with grade IV. The difference between these two proportions explains the statistically significant result. No other factors were significant.

It should be noted that the number of failure events was low and therefore statistical significance is difficult to establish, although the descriptive percentages already showed moderate differences. For example:

Disease duration showed an OR of 1.07 per additional year (95% CI 0.97–1.19; *p* = 0.176). Although the point estimate was above 1, the confidence interval included the null value and no statistically significant association with treatment failure was demonstrated.

Among patients with the most recent diagnosis, only 4.3% were non-responders, compared with 23.5% in the intermediate 3–9-year range and 14.3% among those with long-standing disease (≥10 years). Overall, disease duration of more than 2 years was associated with a less favorable outcome compared with less than 2 years.

Another example concerns target selection: Similarly, the cisternal target showed an OR for nonresponse of 0.24 compared with the retrogasserian target (95% CI 0.03–2.16; *p* = 0.204). Although the point estimate suggested lower odds of failure, the confidence interval was wide and the association was not statistically significant.

**Figure 3:**
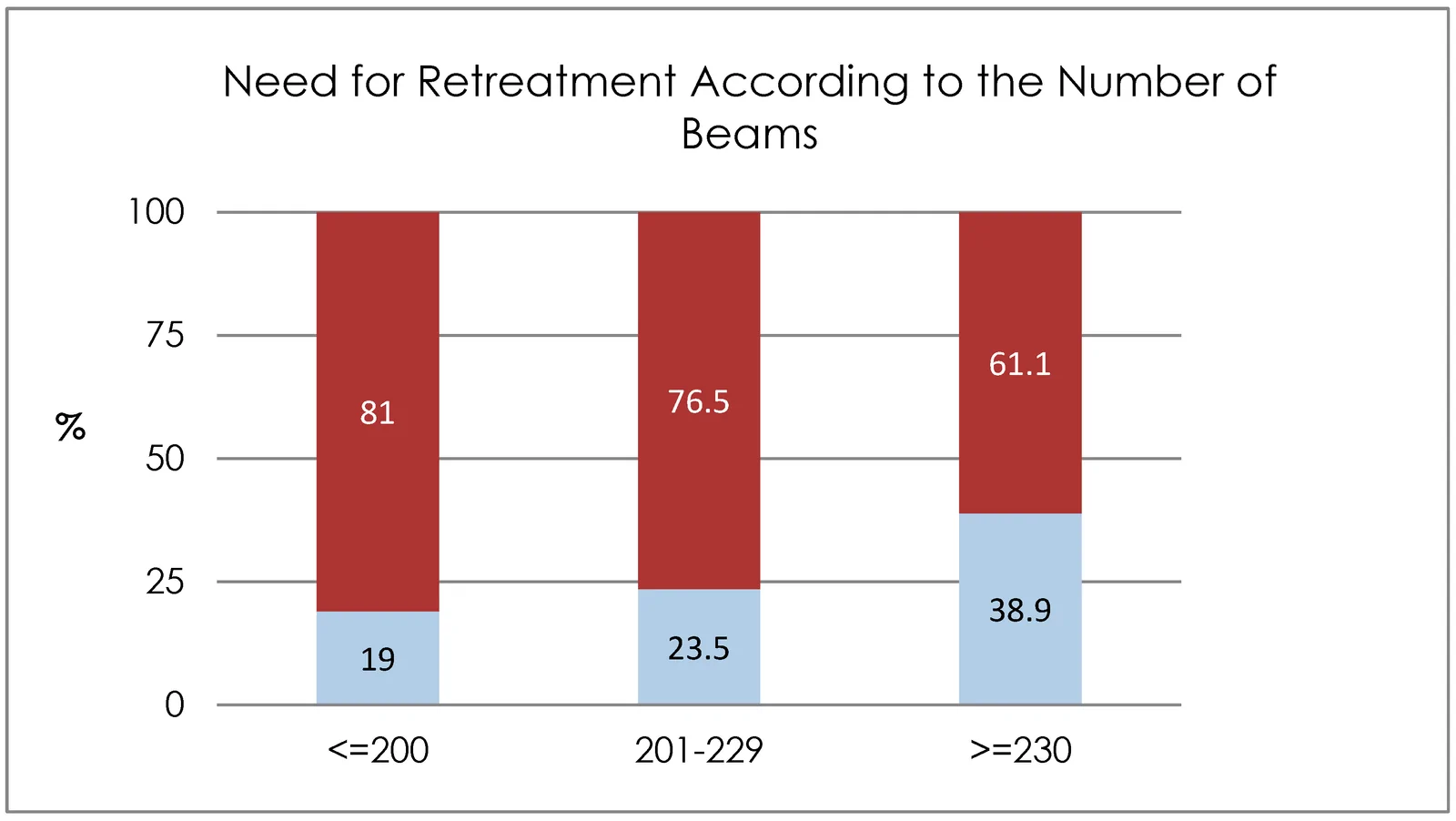
Need for Retreatment According to the Number of Beams. A higher number of arcs used was associated with a greater likelihood of requiring retreatment, as it indirectly reflects an increase in treatment duration.

Regarding the need for retreatment, no significant factors were identified; however, there was a marginal trend involving the number of beams. The greater the number of beams, the greater the probability of retreatment (OR = 1.01; *p* = 0.091) (Figure 3).

No statistically significant association was demonstrated between the number of beams and the need for retreatment. The estimated OR was 1.01 per additional beam (approximately 95% CI 1.00–1.02; *p* = 0.091).

For greater clinical interpretability, this corresponds approximately to an OR of 1.10 per 10 additional beams (95% CI 0.98–1.24), 1.28 per 25 additional beams (95% CI 0.96–1.71), and 1.64 per 50 additional beams (95% CI 0.92–2.93).

Although the point estimates increase as the number of beams is rescaled, all corresponding 95% confidence intervals include the null value. Therefore, no statistically significant association between the number of beams and retreatment was demonstrated, and these findings should be interpreted as exploratory.

No treatment failures were observed among the five patients with MS-associated trigeminal neuralgia. However, the small size of this subgroup precludes reliable comparison with the other etiological groups and does not allow conclusions regarding the effect of multiple sclerosis on treatment response.

## 4. Discussion

### 4.1. Selection of the Radiosurgical Target

In our series, the radiosurgical target was systematically located in the cisternal-retrogasserian portion of the trigeminal nerve, without using the root entry zone (REZ) as the point of maximum dose. Target selection along the cisternal segment remains one of the most debated technical aspects of radiosurgery for trigeminal neuralgia (TN), with two main strategies historically established: a proximal target located at the REZ near the pontine emergence, and a more distal target located in the retrogasserian region [4].

The historical choice of the REZ was based, at least in part, on its particular histological organization. This region contains the transition between central myelin, produced by oligodendrocytes, and peripheral myelin, produced by Schwann cells, corresponding to the Obersteiner–Redlich transition zone. This feature led to the hypothesis that the proximal segment might be more vulnerable both to neurovascular compression and to radiation [16]. However, anatomical studies have demonstrated substantial interindividual variability in the extent of central myelin along the trigeminal nerve, questioning whether this transition can be identified using a fixed anatomical distance from the brainstem [16]. From a dosimetric perspective, moving the isocenter toward the pontine emergence also increases exposure of the REZ and pons to intermediate and high isodoses, which may contribute to a higher incidence of sensory disturbances [4].

Direct clinical comparisons between the two strategies have not consistently demonstrated superiority of the proximal target. Matsuda et al. compared 100 patients treated with a single 4 mm isocenter and found no relevant differences in final pain control between the two locations [17]. Interpretation of their results is nevertheless limited by dosimetric heterogeneity, since the proximal group predominantly received 80 Gy whereas most patients treated in the retrogasserian region received 90 Gy. Park et al. likewise found similar success rates between the retrogasserian region and the REZ (93.8% vs. 87.0%; *p* = 0.631), although time to response was shorter with the retrogasserian target (4.1 vs. 6.4 weeks; *p* = 0.044). Although the overall complication rate was similar, neither bothersome facial hypoesthesia nor dry-eye syndrome was observed in the retrogasserian group, compared with 13.1% and 8.7%, respectively, in the REZ group [18].

The main finding favoring a proximal location comes from the retrospective study by Xu et al., in which 99 patients were treated with a homogeneous maximum dose of 80 Gy [19]. A target close to the brainstem was associated with longer pain relief on Kaplan–Meier analysis (*p* = 0.018), but this greater durability was accompanied by a significant increase in sensory toxicity: BNI II–III facial hypoesthesia occurred in 53% of patients treated proximally versus 25% of those treated with a distal target (*p* = 0.015) [19]. In contrast, Sharim et al., analyzing 67 patients treated with LINAC and an isocenter-to-REZ distance of 0–8.6 mm, found no significant association between this distance and pain relief at 3, 6, 9, or 12 months, nor with the development of facial hypoesthesia [20].

Long-term results obtained with a retrogasserian strategy likewise support the efficacy of this location. Régis et al. analyzed 497 patients treated with a single 4 mm isocenter placed on the cisternal portion of the nerve, at a median distance of 7.6 mm from the pontine emergence [21]. Initially, 91.75% achieved complete pain freedom, with a median time to response of 10 days. The actuarial probabilities of remaining pain-free without medication were 71.8%, 64.9%, 59.7%, and 45.3% at 3, 5, 7, and 10 years, respectively. Although the actuarial incidence of hypoesthesia reached approximately 21% during follow-up, very bothersome facial hypoesthesia was limited to 0.6% [21].

Lovo et al. subsequently provided an additional comparison between the two locations in 49 patients treated with Gamma Knife [22]. When success was defined as BNI I–IIIb, response was significantly higher with the retrogasserian target than with the REZ (81.3% vs. 70.6%; *p* = 0.02). However, the proportion of patients achieving complete pain freedom without medication (BNI I) was numerically higher in the REZ group (47.1% vs. 21.9%), highlighting the influence of the definition used to establish clinical success. Time to response did not differ significantly (22.3 vs. 34.1 days; *p* = 0.277), nor did the overall complication rate (31.3% vs. 35.3%; *p* = 0.773). A particularly relevant aspect was the incorporation of the integral dose received by the nerve: higher integral dose was significantly associated with complications (*p* = 0.02), indicating that outcomes associated with a given target location cannot be interpreted independently of the irradiated nerve volume and total absorbed energy [22].

Overall, the aggregated evidence favors a more distal location. The systematic review of the International Stereotactic Radiosurgery Society (ISRS), which included 65 studies and 6461 patients, found no significant differences between retrogasserian and REZ targets in initial efficacy (*p* = 0.25) or maintenance of pain relief (*p* = 0.18), whereas toxicity was significantly higher with the proximal target (OR 1.78; 95% CI 1.04–3.05; *p* = 0.03) [4]. Based on these findings, the ISRS assigned level II evidence in favor of an anterior target, offering comparable efficacy with a lower risk of sensory toxicity related to proximity to the brainstem [4].

The meta-analysis by Wilson et al. subsequently reinforced this interpretation [23]. After jointly analyzing prescription dose and anatomical location, the authors found that treatment of a distal portion of the nerve was independently associated with better pain control than selecting the REZ as the target (*p* < 0.001), even though median doses delivered distally were higher. No significant differences were observed in the incidence of bothersome hypoesthesia between the two locations [23].

More recently, Warnick et al. reported in a large multicenter cohort that distal targeting was independently associated with a higher probability of initial pain response and longer duration of pain relief [24]. These findings provide additional support for the potential clinical relevance of target location. The radiobiological and dosimetric findings of this study are discussed in greater detail in Section 4.2.

Taken together, the available evidence does not demonstrate a consistent advantage of the REZ as a radiosurgical target. Although some series have reported greater durability of pain relief with proximal locations, this potential benefit has repeatedly been associated with a higher incidence of sensory disturbances. Conversely, ISRS recommendations, subsequent meta-analyses, and more recent multicenter data support the use of a cisternal-retrogasserian target, which can provide at least comparable symptomatic control while limiting brainstem exposure. These findings are consistent with the strategy used in our series and demonstrate that target optimization should consider not only anatomical position but also physical dose, BED, and the volume of nerve included within high-dose regions. This interaction between target location, brainstem exposure, and nerve volume is directly linked to the radiobiological and temporal determinants of irradiation.

### 4.2. Influence of Physical Dose, Dose Rate, BED, and Irradiated Nerve Volume

In the present series, 19.6% of patients experienced clinical improvement within the first 7 days and an additional 32.1% between 7 and 15 days; therefore, slightly more than half of the cohort had improved by 15 days. Because latency was recorded in predefined intervals, the exact median cannot be calculated from these grouped data, although it lies within the 7–15-day interval.

These findings should not be interpreted as evidence of a shorter response latency specific to gyroscopic radiosurgery. In the first clinical ZAP-X series, the median time to pain relief was 15 days, whereas Régis et al. reported a median of 10 days in their 497-patient Gamma Knife series [15,21]. Therefore, comparison of medians does not demonstrate faster clinical response with ZAP-X.

Treatment duration and dose-delivery kinetics remain potentially relevant from a radiobiological perspective because temporal protraction may modify the biologically effective dose through sublethal repair during irradiation. However, patient-specific BED was not directly calculated in the present cohort, and detailed temporal delivery parameters were not available in the anonymized research dataset. Consequently, any relationship between gyroscopic treatment delivery, BED, and latency to pain relief remains hypothetical and cannot be inferred from the present results.

On this basis, optimization of radiosurgery for trigeminal neuralgia (TN) has traditionally focused on the maximum physical dose delivered to the nerve. However, accumulated evidence over the past two decades indicates that clinical response probably does not depend exclusively on this parameter, but rather on the interaction between total dose, the rate and duration of its delivery, and the extent of nerve exposed to different dose regions. This interpretation is particularly relevant in functional radiosurgery, in which exceptionally high single doses are delivered to very small volumes and relatively small changes in treatment geometry or chronology may translate into substantial differences in biologically effective dose (BED) and in the total amount of energy absorbed by the nerve.

The first studies specifically examining the influence of dose rate produced apparently negative results. Balamucki et al. found no significant relationship between dose rate, treatment duration, and pain control in a cohort of patients treated during the decay period of Co-60 sources [25]. Similarly, Arai et al. analyzed 165 patients uniformly treated with 80 Gy using a single 4 mm isocenter and compared dose rates of 1.21–2.05 versus 2.06–3.74 Gy/min. Initial pain relief was 71% and 78%, respectively (*p* = 0.547), with no differences in median duration of control—52 versus 54 months; *p* = 0.403—or in the occurrence of new sensory disturbances—14.5% versus 19.3%; *p* = 0.479—[26]. These findings initially contributed to the view that, within certain ranges, source decay and the consequent prolongation of treatment had limited clinical importance.

Subsequent studies, however, introduced a different interpretation. Lee et al. studied 133 patients treated with a uniform dose of 80 Gy using a single 4 mm isocenter and without sector blocking, thereby reducing some of the main dosimetric confounders [27]. A higher dose rate was associated with earlier pain improvement (*p* = 0.028), while values above 2 Gy/min were associated with a lower probability of recurrence during follow-up (*p* = 0.007). Yang et al. subsequently explored this relationship using the BED concept in 192 patients who likewise received 80 Gy through a single isocenter [28]. Differences in source activity resulted in dose rates of 1.19–2.86 Gy/min and approximate irradiation times of 28–69 min. In this setting, with physical dose kept constant, BED was an independent predictor of clinical improvement, and a 1.5 Gy/min decrease in dose rate was associated with a 31.8% reduction in overall improvement in pain intensity. The potential radiobiological benefit of higher BED values was nevertheless accompanied by a higher incidence of hypoesthesia in the highest exposure quartile [28].

BED allows these findings to be integrated from a radiobiological perspective. Unlike physical dose, it incorporates repair of sublethal damage occurring during radiation delivery. For the same prescribed dose, prolongation of exposure provides more opportunity for repair during treatment, reducing the biologically effective component of irradiation; conversely, more temporally concentrated delivery limits this repair. In intracranial radiosurgery, linear-quadratic models modified using protraction factors have been used, followed by biexponential repair models accounting for fast and slow components of the repair process [29,30]. In the main TN studies, an α/β ratio of 2.47 Gy and approximate repair half-times of 0.19 and 2.16 h have commonly been used; these parameters derive from experimental models of central nervous tissue and should be regarded as a radiobiological approximation applied to the trigeminal nerve, rather than values specifically determined for this nerve [29].

The clinical relevance of this approach was demonstrated by Tuleasca et al. in 408 patients treated with Gamma Knife with physical doses of 75–97.9 Gy delivered over 25–135 min [29]. Whereas physical dose was not significantly associated with hypoesthesia, a progressive relationship was observed with BED: the incidence of sensory disturbance increased from less than 5% at approximately 1800 Gy_2.47_ to approximately 42% at values close to 2600 Gy_2.47_. Pain control at one and two years remained around 90% across a broad BED range, although patients exposed to lower values reached the pain-free state more slowly. The authors identified an approximate range of 1820–1962.5 Gy_2.47_ associated with a high probability of efficacy and relatively low incidence of hypoesthesia [29]. Rather than establishing a universal threshold, these results introduced the concept of a possible radiobiological therapeutic window, in which increasing biological effect could accelerate or intensify the response up to a point beyond which sensory toxicity would predominate.

The most recent multicenter evidence supports this interpretation but highlights that BED and physical dose cannot be considered independently of target location and the spatial distribution of irradiation. Warnick et al., in a cohort of 871 patients treated as a first radiosurgical procedure, observed an initial BNI I–IIIa response in 81.8%, with a median of 30 days to relief [24]. For distal targets, BED ≥ 2100 Gy_2.47_ was independently associated with a higher probability of relief at 30 days (HR 1.46; 95% CI 1.05–2.03; *p* = 0.03) and at one year (HR 1.20; 95% CI 1.00–1.44; *p* = 0.049), whereas for proximal locations physical dose ≥ 85 Gy showed greater predictive value. In parallel, a maximum point dose to the brainstem ≥ 29.5 Gy was associated with a higher probability of BNI Facial Numbness III [24]. These findings indicate that the same BED value does not necessarily produce the same clinical effect when the anatomical region of the nerve receiving the dose changes.

Tang et al. expanded this concept through a multicenter study of 548 patients, moving beyond consideration of BED as a single maximum value to analyze its distribution using BED-volume histograms [31]. The proportion of the trigeminal nerve included within the 1000 Gy_2.47_ iso-BED was associated both with response during the first month and with subsequent recurrence. In addition, the combination of maximum brainstem BED and iso-BED coverage of the nerve segment adjacent to the REZ predicted facial hypoesthesia. These results are particularly relevant because they integrate the magnitude of irradiation, its temporal component, and the anatomical volume on which it acts within the same model, moving beyond interpretation based exclusively on maximum point dose [31].

The most recent synthesis of the evidence comes from the meta-analysis by Jomy et al., which included eight cohorts and 2596 patients [32]. Beam-on times ranged from 27 to 171 min, physical doses from 62.5 to 95 Gy, median BED was approximately 2105 Gy_2.47_, and median dose rate was 2.2 Gy/min. Higher dose rates were associated with a significantly greater probability of pain relief (RR 1.36; 95% CI 1.10–1.67; *p* = 0.005). However, meta-regression using dose rate as a continuous variable did not demonstrate a significant linear relationship (β = 0.26; 95% CI −1.09 to 1.60; *p* = 0.71), and overall certainty of evidence was considered low or very low because the cohorts were predominantly retrospective and the radiobiological models were heterogeneous [32]. Therefore, aggregated data support an influence of delivery kinetics, but do not yet allow definition of a universal dose-rate or BED value that can be directly transferred across different platforms and techniques.

The temporal dimension of irradiation must be interpreted together with its spatial dimension. One of the key studies in this regard was the prospective, randomized, double-blind trial by Flickinger et al., in which 87 patients were treated with 75 Gy using one or two 4 mm isocenters [33]. Increasing the irradiated nerve length through a second isocenter did not improve pain control, whereas the occurrence of hypoesthesia and paresthesias was significantly correlated with the length of nerve included in the irradiated volume (*p* = 0.018). This phenomenon, frequently termed the Flickinger effect, is one of the main pieces of evidence that indiscriminately increasing the volume of exposed nerve may enlarge the functional lesion without necessarily providing additional analgesic benefit [33].

The importance of irradiated volume has been confirmed through different dosimetric approaches. Massager et al. evaluated the effect of selective beam-channel plugging in 109 patients treated with 90 Gy [34]. Although blocking was used to limit brainstem exposure, it paradoxically produced a redistribution of dose that increased the length of nerve exposed to high-dose regions. Mean trigeminal dose was significantly higher when blocked channels were used—42.86 versus 38.01 Gy; *p* = 0.002—as was integral dose—3.35 versus 3.12 mJ; *p* = 0.027. The proportion of excellent analgesic outcomes was higher (84% vs. 62%), but sensory toxicity also increased, with bothersome hypoesthesia in 10% versus 2% of patients [34]. Thus, a maneuver intended geometrically to protect an adjacent structure may simultaneously alter the longitudinal dose distribution along the nerve, highlighting the limitations of evaluating a plan solely by maximum dose and brainstem dose.

Consistently, Zhang et al. specifically analyzed mean dose, integral dose, and irradiated trigeminal root volume in 73 patients [35]. A higher integral dose to the nerve did not significantly improve pain control, whereas it was associated with increased trigeminal toxicity (*p* = 0.005). Lovo et al. likewise incorporated integral dose into their analysis of proximal and retrogasserian targets and found a significant association between greater energy deposited in the nerve and the occurrence of complications (*p* = 0.02) [22]. Although these studies differ methodologically and do not allow a specific volumetric threshold to be defined, their findings converge with the Flickinger trial in indicating that exposure of a greater amount of neural tissue cannot be assumed to be equivalent to greater efficacy.

Taken together, these observations suggest that the radiosurgical response of TN is conditioned by a true spatiotemporal dose distribution. Physical dose defines the amount of deposited energy; treatment time and dose rate modify the degree of sublethal repair and therefore the BED; and plan geometry determines what length and volume of the nerve are exposed to each dose level or iso-BED region. From this perspective, two treatments with identical maximum doses may not be radiobiologically equivalent if they differ in duration, dose rate, target location, irradiated nerve volume, or isodose distribution. The results of Warnick and Tang currently provide the most direct clinical evidence of this interaction between anatomical location, BED, and exposed volume [24,31].

This interpretation is particularly relevant when analyzing dedicated LINAC systems with delivery characteristics different from Gamma Knife. ZAP-X uses a 3-MV S-band linear accelerator with a nominal output rate of 1500 MU/min [7]. Importantly, these 1500 MU/min cannot be directly equated with the Gy/min values reported in Co-60 studies, because the effective absorbed dose rate in the nerve depends on the collimator used, its output factors, the number of monitor units, beam weighting, and the overall plan geometry. Therefore, any potential radiobiological advantage associated with high nominal delivery capacity cannot be inferred from the MU/min value alone.

The first dosimetric study specifically devoted to TN with ZAP-X also illustrates differences in radiation delivery compared with a conventional isocentric system. Romanelli et al. used two isocenters, 81 beams, and 29,441 MU, with an estimated treatment time of approximately 25 min [8]. Subsequently, in the first clinical series of gyroscopic radiosurgery for TN, 30 patients received 90 Gy to an approximately 5 mm retrogasserian segment using a single isocenter; planning used a median of 221 beams and median treatment time was 37 min [15]. In this cohort, median BNI decreased from IV to II, 80% achieved pain freedom or occasional pain without medication, and median time to relief was 15 days, although these early clinical results do not establish a causal relationship between treatment temporal structure and speed of response [15].

The possible influence of the number of beams or trajectories has gained interest from recent technical studies of ZAP-X. Chaga et al., using prospective data from 200 patients, identified gantry movement time and the number of beams and isocenters among the factors with the greatest influence on procedure duration [36]. The regression model explained 98.4% of the variability in treatment time. In the subsequent clinical experience of 200 patients published by Chen et al., which included 18 TN cases, it was confirmed that ZAP-X delivery comprises temporally distinct components related to beam-on, system movements, image acquisition and processing, and repositioning [37].

These results allow a relevant radiobiological hypothesis to be proposed for TN planning with ZAP-X. If an increase in the number of beams or arcs prolongs the interval between the first and last exposure while physical dose remains constant, the sublethal repair model predicts a reduction in BED compared with more temporally concentrated delivery [29,30]. This potential relationship does not depend only on cumulative beam-on time, but on the complete temporal pattern once irradiation has begun, because repair continues during the intervals between exposures. Consequently, two plans delivering the same maximum dose and a comparable number of MUs may have different radiobiological efficacy if the beam sequence and intervening periods result in substantially different effective delivery times.

This interpretation nevertheless requires differentiation between total procedure time and radiobiologically relevant time. Positioning or checks performed before the first beam do not modify BED; by contrast, gantry movements, isocenter changes, image verifications, or repositioning occurring between exposures may contribute to sublethal repair. Therefore, using only the time recorded from patient entry into the machine until completion of the procedure may introduce substantial error into the radiobiological estimate. The models developed by Jones and Hopewell specifically allow treatment time and intervals between exposures to be incorporated into BED calculation [30].

Within this framework, the number of arcs could be interpreted as an indirect marker of plan temporal complexity, but not as an independent biological determinant. A greater number of trajectories may favor a more conformal spatial dose distribution and modify the volume of nerve included within high- and intermediate-dose regions; simultaneously, it may increase the number of movements and the elapsed delivery time. These two effects could act in potentially different directions: more complex geometry may optimize dose distribution, whereas greater temporal protraction may reduce BED for a constant physical dose. The currently available ZAP-X literature demonstrates an association between plan complexity and treatment duration [36,37], while the clinical GKRS literature demonstrates an association between time/dose rate, BED, and clinical response [24,28,29,30,31,32].

This integration of spatial and temporal components may be particularly useful for interpreting differences in response among patients treated with identical maximum doses. Prescribed dose remains an essential parameter, but available data indicate that it is insufficient to fully characterize irradiation received by the trigeminal nerve. Joint incorporation of BED, effective dose rate, duration from the first to the last beam, nerve length and volume exposed, mean dose, integral dose, and dose/BED-volume histograms provides a more complete characterization of treatment. The most recent evidence, particularly the multicenter studies by Warnick and Tang and the Jomy meta-analysis, supports this transition from planning based primarily on point dose toward radiobiological and volumetric evaluation of the nerve [24,31,32].

From this perspective, the therapeutic balance of TN radiosurgery does not appear to depend on maximizing any single parameter in isolation. Increasing BED may favor a faster response or a higher probability of relief but may also increase sensory disturbances; increasing nerve length or integral dose may broaden the functional effect, but randomized evidence and dosimetric studies show that this increase may translate predominantly into toxicity [22,29,33,34,35]. Radiosurgical optimization should therefore be understood as the search for a spatial dose distribution sufficiently effective over the pain-generating segment while simultaneously maintaining a temporal delivery pattern that preserves the desired biological effect without exceeding the functional tolerance of the nerve and adjacent structures. In gyroscopic platforms such as ZAP-X, joint analysis of these parameters may be particularly relevant because of the use of numerous beams and trajectories and the temporal separation between different exposures.

### 4.3. Radiosurgery for Trigeminal Neuralgia Secondary to Multiple Sclerosis

Trigeminal neuralgia secondary to multiple sclerosis (MS-TN) represents a particularly complex setting for radiosurgery because of the potential coexistence of cisternal nerve involvement and demyelinating lesions affecting central trigeminal pathways. Published evidence over the last decade, primarily from Gamma Knife radiosurgery (GKRS) series, shows that radiosurgery can achieve satisfactory initial pain control in a high proportion of patients, although durability is considerably more variable. In one of the largest available multicenter series, Xu et al. analyzed 263 patients with MS-TN and observed an initial BNI I–IIIb response in 88.2%, with a median time of approximately one month to onset of relief. However, the probability of maintaining satisfactory control progressively declined to 54% at one year, 35% at two years, and 24% at four years [38]. Similarly, Przybylowski et al., with a median follow-up of 78 months, reported pain-control rates of 62%, 29%, 22%, and 13% at 1, 3, 5, and 7 years, respectively [39]. These findings indicate that, in MS-TN, the high initial response rate should be interpreted separately from the durability of radiosurgical benefit.

Recent meta-analyses confirm this temporal evolution. Spina et al., including 12 studies and 646 patients, estimated a pooled initial response rate of 83% (95% CI 74–90%), which decreased to 47% (95% CI 33–60%) at the end of follow-up; moreover, follow-up duration was the only variable significantly associated with decreasing long-term success in meta-regression (β = −0.0051; *p* = 0.0047) [40]. Subsequently, Ali et al. obtained similar results, with a pooled initial response of 83%, overall control during follow-up of 51%, and a recurrence rate close to 40% [41]. The most recent meta-analysis, by Hajikarimloo et al., including 23 studies and 837 patients, also introduced a clinically relevant distinction between complete pain freedom and adequate relief: initially, 32% of patients achieved complete pain freedom and 74% achieved satisfactory relief, whereas at the end of follow-up these figures were 17% and 65%, respectively [42].

The maximum dose used in most contemporary series ranges from 80 to 90 Gy, usually delivered through a single 4 mm isocenter on the cisternal segment of the trigeminal nerve. However, there is currently insufficient evidence to establish an optimal dose or target location specifically for MS-TN. Helis et al., in a cohort of 77 patients, did not identify dosimetric parameters significantly related to duration of relief after first GKRS, and the presence of an ipsilateral pontine plaque did not predict treatment failure either [43]. These findings suggest that the relationship between dose distribution, anatomy of the demyelinating lesion, and clinical response is probably more complex than simple proximity of the target to the root entry zone. Experiences using different radiosurgical geometries also support the potential relevance of irradiated nerve length and volume. Conti et al., targeting an approximately 4–6 mm retrogasserian segment, achieved a BNI I–IIIb response in 85% during the first weeks, although only 44% maintained control at four years [44]. The heterogeneity of these strategies currently prevents determination of whether maximum dose, irradiated length, integral dose, or specific dose-volume metrics are better predictors of outcome.

Radiosurgery-related toxicity is predominantly sensory. The meta-analysis by Ali et al. estimated an incidence of facial hypoesthesia of 19.6%, although only 4.1% experienced a sensory disturbance considered bothersome [41]. This distinction is relevant because the presence of any degree of hypoesthesia does not necessarily equate to clinically significant toxicity. Helis et al. observed a 26.3% incidence of hypoesthesia after first GKRS, but only 4.1% of patients considered it bothersome [43]. Therefore, safety assessment should systematically distinguish any sensory alteration from clinically consequential toxicity, preferably using standardized scales such as the BNI Facial Numbness Scale.

Pain recurrence has also driven the development of repeat radiosurgery strategies. In the series by Helis et al., a second GKRS achieved BNI IIIb or better in 88% of patients, with a median duration of relief of 4 years and control probabilities of 70% and 54% at 1 and 3 years, respectively [43]. Przybylowski et al. likewise observed that retreatment could prolong the cumulative period of control, although efficacy again declined with prolonged follow-up [39]. More recently, Franzini et al. reported BNI IIIa or better in 15 of 18 patients undergoing a second GKRS, with a median time to improvement of 21 days and a 50% probability of maintaining control beyond 3 years [45]. The main limitation of retreatment appears to be cumulative sensory toxicity: Helis et al. observed hypoesthesia after a second irradiation in 78.8% of cases, although only 2.9% considered it bothersome, with associations between cumulative dose to different structures and the occurrence of sensory disturbances [43].

Overall, current evidence indicates that radiosurgery provides a high initial response rate in MS-related TN, generally between 70% and 85%, with a therapeutic latency usually of several weeks, but with progressive loss of control during follow-up and a substantial proportion of recurrences. Repeat treatment can restore benefit in a relevant proportion of patients, although it increases the frequency of sensory disturbances. The main limitations of the available evidence are its predominantly retrospective nature, heterogeneity in response and recurrence definitions, and the absence of dosimetric standardization specific to MS-TN. Prospective studies are needed to determine which dose parameters, target locations, and irradiated nerve volumes are associated with more durable responses, as well as to establish homogeneous criteria for clinical and toxicity assessment that allow reliable comparison of radiosurgical outcomes.

### 4.4. Radiosurgery for Trigeminal Neuralgia Secondary to Tumor Compression and Neurovascular Conflict

Trigeminal neuralgia secondary to space-occupying lesions represents a minority but clinically relevant group, in which skull-base meningiomas, vestibular schwannomas, and trigeminal schwannomas are the most frequent tumor etiologies. In these patients, the pain mechanism may be related to direct compression and deformation of the nerve, infiltration or displacement, or even secondary neurovascular compression produced by tumor-induced anatomical distortion. This circumstance conceptually distinguishes these cases from other forms of neuralgia, because treatment may be directed exclusively at the trigeminal nerve, the causal lesion, or both simultaneously.

Stereotactic radiosurgery has demonstrated the ability to provide both tumor control and pain relief, although these two effects do not necessarily evolve in parallel. The meta-analysis by Peciu-Florianu et al., focused mainly on meningiomas and vestibular schwannomas, showed complete pain freedom (BNI I) in approximately 50.5% of patients and a BNI I–IIIb outcome in 83.8%, with recurrence rates close to 35% and an overall complication rate of 12.6%, predominantly facial sensory disturbances [46]. More recent pooled evidence provides somewhat more conservative estimates. Hajikarimloo et al. analyzed 19 studies and 454 patients and estimated a pooled rate of complete pain freedom of 38% (95% CI 27–50%), adequate relief of 73% (95% CI 63–83%), and radiation-related adverse effects of 14% (95% CI 7–22%) [47]. In the petroclival meningioma subgroup, complete pain freedom decreased to 30% and adequate relief to 64%, demonstrating that excellent radiological tumor control does not necessarily guarantee resolution of neuralgia.

The optimal irradiation strategy is also not definitively established. Radiosurgery directed exclusively at the tumor aims to control the lesion and progressively reduce its effect on the nerve, whereas dual treatment incorporates a high functional dose to the trigeminal nerve. However, available meta-analyses have not consistently demonstrated superiority of simultaneous tumor-and-nerve treatment over tumor-only treatment [46,47]. In a contemporary series of 44 patients treated with simultaneous irradiation of the tumor and trigeminal nerve, Nguyen et al. observed significant pain improvement in 86.4%, with a median latency of 2 months; however, 36.4% experienced recurrence and only 50% maintained durable relief after a median follow-up of 31.9 months. In addition, 31.8% developed a treatment-related adverse effect and 6.8% developed permanent facial hypoesthesia [48]. These data suggest that extending irradiation to the nerve may enhance the functional component of treatment, but its potential benefit must be weighed against increased trigeminal exposure and sensory toxicity.

Nevertheless, the presence of an anatomical lesion responsible for frank compression requires consideration of whether radiosurgery should truly constitute primary treatment. Available evidence favors surgical decompression when the lesion can be resected with acceptable risk. In a systematic review of cerebellopontine angle tumors, Nugroho et al. found pain improvement in 92.2% of patients treated with resection versus 79.1% after tumor-directed radiosurgery, with recurrence rates of 4.5% and 26.5%, respectively [49]. These results were confirmed by subsequent analyses. Hallak et al., specifically in petroclival meningiomas, observed significantly greater pain resolution after resection than after radiosurgery (82% vs. 31%; *p* = 0.004), as well as lower persistence (0% vs. 25%; *p* = 0.001) and pain exacerbation (0% vs. 12%; *p* = 0.001) [50]. Consistently, the most recent meta-analysis by Penchev et al., which included 32 studies and 662 patients with cerebellopontine angle tumors, confirmed that surgical resection was associated with a significantly greater probability of pain relief (*p* = 0.0009) and lower recurrence (*p* = 0.0267), without significant differences in the incidence of facial hypoesthesia (*p* = 0.5306) [51].

Therefore, when neuralgia represents the manifestation of clearly identifiable and surgically correctable mechanical tumor compression, current evidence favors decompression of the trigeminal root by treating the causal lesion as the first strategy in appropriately selected patients. Primary radiosurgery is particularly relevant, however, in small or moderate-sized, benign, radiosensitive tumors in which it would already be a reasonable option for tumor control itself, as well as in elderly patients, those with significant comorbidity, lesions in surgically complex locations, patients at high risk of cranial nerve deficits, or those who decline open surgery. In these circumstances, the objective should jointly encompass lesion control and pain relief, avoiding consideration of nerve-only irradiation as an automatic substitute for etiological treatment.

A different consideration applies to patients with primary neurovascular conflict and morphological changes in the trigeminal nerve, in the absence of a causal tumor lesion. Although radiosurgery can achieve satisfactory outcomes even when vascular compression is present—Chang et al. found no significant differences in pain control among patients without neurovascular conflict, with compression by small vessels, or with compression by large vessels (85%, 75%, and 88%, respectively; *p* = 0.289) [52]—demonstrated vascular compression changes the therapeutic hierarchy. The European Academy of Neurology guidelines recommend microvascular decompression as the procedure of choice in patients with classical TN and neurovascular conflict accompanied by morphological changes, provided the patient can undergo posterior fossa surgery [3]. This recommendation remains consistent with the most recent comparative evidence: the updated meta-analysis by Fonseca et al., including 8444 patients, found a greater probability of pain relief with microvascular decompression than with Gamma Knife (RR 1.87; 95% CI 1.38–2.53; *p* < 0.001) and a significantly lower risk of recurrence (RR 0.32; 95% CI 0.14–0.74; *p* = 0.008), at the cost of a higher overall incidence of surgical complications (RR 3.37; 95% CI 1.39–8.15; *p* = 0.007) [53].

Accordingly, radiosurgery can be considered an effective primary treatment in selected patients with neuralgia associated with tumor or vascular lesions, but demonstration of a compressive cause amenable to direct correction should form part of the therapeutic decision. In small, difficult-to-access benign tumors, primary irradiation of the lesion may simultaneously provide tumor control and pain improvement with limited morbidity; conversely, when tumors cause significant compression and are reasonably resectable, evidence favors surgical decompression. Similarly, in classical TN with morphologically significant neurovascular conflict, radiosurgery is a valid alternative when open surgery carries excessive risk or is not accepted by the patient, but current evidence does not justify systematically displacing microvascular decompression as the primary strategy.

## 5. Conclusions

In the clinical series analyzed by the principal investigator, treatment of TN with gyroscopic radiosurgery produced outcomes during the minimum 12-month follow-up period that were similar to the satisfactory outcome rates reported in series based on Cobalt-60 platforms.

In our series, consistent with more recent publications, MS-related TN was not a factor associated with treatment non-response, nor was it associated with a longer latency to clinical response, as no statistically significant differences were found in subgroup analyses and MS was not identified as a predictor of non-response. The same was true for the presence of associated tumor or vascular pathology. However, female sex trended towards a favorable prognostic factor in our sample. Although the point estimate suggested lower odds of treatment failure in women (OR 0.65; 95% CI 0.13–3.24), the association was not statistically significant (*p* = 0.596), and the available data do not support considering female sex a prognostic factor, whereas the literature has generally reported sex as a factor without an impact on treatment response.

Consistent with previous publications establishing that prolonged treatment time is associated with a higher risk of treatment failure or a shorter pain-free period, our study demonstrates that a greater number of arcs is associated with a higher probability of requiring retreatment. This may be indirectly related to the number of arcs and the intervening times they entail (new positioning and IGRT), which prolong treatment. Although statistical significance was not reached, this finding should prompt consideration of using more distal targets relative to the REZ, which usually involves less complex planning and a lower requirement for multiple isocenters, or, in Co-60-based systems, source blocking.

## 6. Limitations

This study has several limitations that should be considered when interpreting its findings. First, it represents a single-center retrospective series with a relatively small sample size and only seven treatment failures, which limits statistical power and the precision of effect estimates, particularly for analyses of potential prognostic factors. The cohort was also clinically heterogeneous with regard to trigeminal neuralgia etiology and previous treatments, while some etiological subgroups, particularly MS-associated trigeminal neuralgia, contained very few patients and therefore do not permit reliable subgroup comparisons. Follow-up duration was not identical for all patients, potentially affecting assessment of recurrence and durability of pain control. In addition, the absence of a contemporaneous control group precludes direct comparison with alternative radiosurgical platforms or treatment strategies. Several clinical and technical variables were explored statistically, increasing the possibility of chance findings arising from multiple comparisons; consequently, these analyses should be regarded as exploratory and hypothesis-generating. Target location was individualized according to anatomical and dosimetric considerations rather than randomly assigned, introducing potential selection bias when comparing cisternal and retrogasserian targets. Finally, although the number of beams and other treatment-planning variables were available, biologically effective dose was not directly calculated and detailed temporal delivery parameters, including total treatment time and beam-on time, were not systematically available. Therefore, the potential radiobiological relationship between treatment duration, BED, dose distribution, and clinical outcome could not be directly evaluated in this cohort.

## Figures and Tables

**Table 1:** Patient profile and intervention characteristics according to treatment outcome (final BNI): number of patients (%) or mean ± standard deviation.

	SUCCESS	FAILURE
TOTAL	49 (100%)	7 (100%)
**SEX (men)**	16 (32.7)	3 (42.9)
**AGE**	65.4 ± 14.9	69.4 ± 11.6
**SUBTYPE**		
Classical	1 (2.0)	0 (0.0)
Secondary	8 (16.3)	0 (0.0)
Multiple sclerosis	5 (10.2)	0 (0.0)
Idiopathic	35 (71.4)	7 (100)
**DISEASE DURATION (years)**	6.2 ± 6.7	10.0 ± 6.7
**PREVIOUS TREATMENT**		
Medication only	26 (53.1)	2 (28.6)
Medication + infiltration	2 (4.1)	1 (14.3)
Medication + intervention	13 (26.5)	2 (28.6)
Medication + infiltration + intervention	8 (16.3)	2 (28.6)
**NUMBER OF BEAMS**	211.0 ± 76.4	226.1 ± 63.3
**COLLIMATOR**		
4 mm	46 (90.2)	5(9.8)
5 mm	3 (60)	2 (40)
**NERVE VOLUME**		
0.02 cc	12 (24.5)	2 (28.6)
0.03 cc	19 (38.8)	3 (42.9)
0.04 cc	11 (22.4)	1 (14.3)
0.05 cc	7 (14.3)	1 (14.3)
**TARGET**		
Retrogasserian	29 (59.2)	6 (85.7)
Cisternal	20 (40.6)	1 (14.3)
Baseline BNI (T0)		
IIIa	1 (2.0)	0 (0.0)
IV	42 (85.7)	3 (42.9)
V	6 (12.2)	4 (57.1)

**Table 2:** Post-treatment BNI outcome according to sex.

Outcome	Total N	Total %	Male N	Male %	Female N	Female %
Total	56	100.0	19	100.0	37	100.0
Success	49	87.5	16	84.2	33	89.2
Failure	7	12.5	3	15.8	4	10.8

**Table 3:** Failure (non-response to treatment) according to patient profile and intervention characteristics: results of a simple binary logistic regression model, unadjusted OR and 95% confidence interval. Chi-square test result when the OR could not be estimated.

	OR	95% CI	*p*-Value
**SEX**			
Male	1		
Female	0.65	0.13–3.24	0.596
AGE	1.02	0.96–1.08	0.497
**SUBTYPE**	--	--	0.446 (Chi2)
Classical	--	--	--
Secondary	--	--	--
Multiple sclerosis	--	--	--
Idiopathic	--	--	--
**DISEASE DURATION (years)**	1.07	0.97–1.19	0.176
**PREVIOUS TREATMENT**			0.535
Medication only	1		
Medication + infiltration	6.50	0.39–106.7	0.190
Medication + intervention	2.00	0.25–15.8	0.512
Medication + infiltration + intervention	3.25	0.39–26.9	0.275
NUMBER OF BEAMS	1.00	0.99–1.01	0.614
**COLLIMATOR**			
4 mm	1		
5 mm	0.48	0.0–2.45	0.378
**NERVE VOLUME**			0.969
0.02 cc	1		
0.03 cc	0.95	0.14–6.53	0.956
0.04 cc	0.55	0.04–6.89	0.639
0.05 cc	0.86	0.07–11.3	0.907
**TARGET**			
Retrogasserian	1		
Cisternal	0.24	0.03–2.16	0.204
Baseline BNI (T0)			
IIIa	--	--	--
IV	1		
V	9.33	1.66–52.3	0.011 *

* *p* < 0.05.

**Table 4:** Treatment success according to pre-treatment BNI score (T0).

Outcome	Total N	Total %	BNI IIIa N	BNI IIIa %	BNI IV N	BNI IV %	BNI V N	BNI V %
Total	56	100.0	1	100.0	45	100.0	10	100.0
Success	49	87.5	1	100.0	42	93.3	6	60.0
Failure	7	12.5	0	0.0	3	6.7	4	40.0

## Data Availability

The data presented in this study is available upon request from the corresponding author. The data are not publicly available due to patients’ privacy.
